# A zeolite templating method for fabricating edge site-enriched N-doped carbon materials†

**DOI:** 10.1039/d3na00186e

**Published:** 2023-05-12

**Authors:** Yurika Taniguchi, Yasuhiro Shu, Ryuji Takada, Koji Miyake, Yoshiaki Uchida, Norikazu Nishiyama

**Affiliations:** a Division of Chemical Engineering, Department of Materials Engineering Science Graduate School of Engineering Science, Osaka University 1-3 Machikaneyama Toyonaka Osaka 560-8531 Japan kojimiyake@cheng.es.osaka-u.ac.jp; b Innovative Catalysis Science Division, Institute for Open and Transdisciplinary Research Initiatives (ICS-OTRI), Osaka University Suita Osaka 565-0871 Japan

## Abstract

N-doped carbon materials have attracted considerable attention as highly functional materials because nitrogen doping distorts the carbon lattice, changes the charge density, and introduces additional defects. Among various positions of N atoms in N-doped carbon compounds, pyridinic-N, pyrrolic-N, and valley-N, which are doped at edge sites, exhibit specific electrocatalytic activities during the oxygen reduction reaction (ORR). However, it is difficult to selectively introduce these N atoms into a carbon matrix because the synthesis procedure typically includes high-temperature heat treatment. In this study, we applied a zeolite templating method to synthesize edge site-rich N-doped carbon materials. The sample fabricated using a zeolite template possessed high concentrations of pyridinic-N and valley-N atoms, demonstrating a significantly higher ORR catalytic activity than the sample synthesized without a zeolite template. Additional experiments conducted using various zeolites confirmed the positive effect of N-doped carbons on the ORR catalytic performance. This work demonstrated that the zeolite templating method not only increased the specific surface area and the number of active sites but also selectively created edge sites and improved the quality of the active sites.

## Abstract 2

This study proposes a zeolite templating method for manufacturing N-doped carbon materials that enable precise control of the locations of catalytically active N sites for the ORR-190546672500.

## Graphical abstract

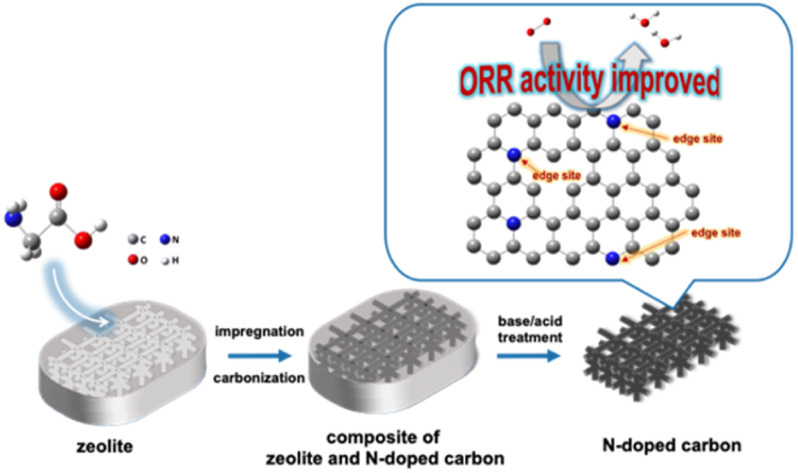

## 1 Introduction

Carbon materials have a very long history. Until the 1950s, carbon black^1^ and activated carbon^2^ used as electrode materials were actively studied by researchers. In the 1960s, carbon fibers^3^ and glass-like carbon^4^ were synthesized, and their application scope expanded rapidly to include biomaterials, fuel cells, and nuclear fusion reactor wall materials. In the 1990s, nanocarbons, such as fullerenes described by the formula C_60_ (ref. 5) and nanotubes,^6^ spread rapidly around the world. Various studies on carbon materials, including graphene, are currently ongoing. Carbon materials are also applied as the main electrode materials in commercial lithium-ion batteries^7^ and supercapacitors^8,9^ because of their high electrical conductivity, chemical stability, low fabrication cost, and environmental friendliness.^10–12^ Although carbon materials are utilized in many fields, pure carbon is not always practically applicable, and its performance can be considerably enhanced by chemical modifications, such as doping with heteroatoms (including N, P, B, and S).^11,13^

Various types of heteroatom-doped carbon materials (HDCMs), ranging from those doped with a single heteroatom to those doped with a combination of several heteroatoms, have been examined over the past few years.^12,14–16^ Because the sizes and electronegativities of heteroatoms differ from those of C, HDCMs exhibit structural distortion and charge density variations. In addition, the number of catalytically active sites can be regulated by introducing heteroatoms or lattice defects, while the covalent bonds formed between carbon atoms and heteroatoms increase material stability.^12^ These characteristics of HDCMs enable their applications in the oxygen reduction reaction (ORR), batteries, and semiconductors.

Among various HDCMs, nitrogen-doped carbon materials (NDCMs) are remarkable materials that have been extensively studied over the past few decades and can be potentially utilized in many prospective areas.^17,18^ These include fuel cells, solar cells, lithium-ion batteries,^19^ supercapacitors, chemical catalysts, and catalyst supports.^10,20^

The introduction of a nitrogen atom with an electronegativity different from a carbon atom into the graphene lattice breaks the electrical neutrality of graphene. It creates charged sites favorable for the oxygen adsorption during the ORR in addition to defect formation,^21^ which increases the electrical conductivity and oxygen reduction capacity of the material.^22,23^ Although platinum catalysts are mainly used as catalysts in fuel cells currently, by increasing the effectiveness of NDCMs as ORR catalysts, it may be possible to develop new catalysts with lower fabrication costs and higher durability than those of platinum catalysts.^24–27^ Many researchers have started investigating NDCMs since the discovery of NDCMs for the ORR in 2009.^22^

The advantages and disadvantages of doping heteroatoms vary depending on the heteroatom type, and a rational design is usually required to utilize their advantages. There are two main methods for synthesizing NDCMs.^13,24,25^ The first method is calcinating compounds with high contents of heteroatoms under an inert atmosphere, which are doped during carbonization. The second one is a post-synthetic method that introduces heteroatoms into carbon materials using reactive heteroatom sources. This method has been reported in the literature^28,29^ but not extensively studied.

**Fig. 1:**
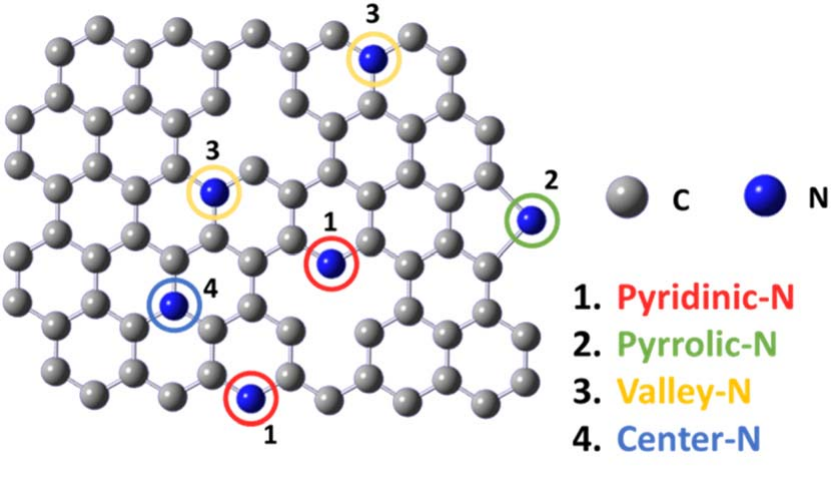
Various doping configurations of nitrogen atoms in the graphite structure (1. pyridinic-N, 2. pyrrolic-N, 3. valley-N and 4. center-N).

In previous studies, the active sites of NDCMs^21^ were identified by computational calculation. Depending on the nitrogen dopant position, four different types of N-doped carbon sites exist:^13,23,30^ pyridinic-N, pyrrolic-N, valley-N, and central-N (Fig. 1). Among these types, pyridinic-N, pyrrolic-N, and valley-N are the suitable active sites,^12,23,30,31^ which adjust the electronegativity of the neighboring carbons to the optimum properties for the ORR. Therefore, it is highly desirable to develop synthetic methods that can selectively produce pyridinic-N, pyrrolic-N, and valley-N sites.^32,33^ Generally, the active sites located at the graphene edge were considered owing to high activity. Therefore, the activity of NDCMs can be considerably improved by doping the edge sites with nitrogen.^34,35^

Until recently, it has been difficult to synthesize such materials while controlling active site locations because the preparation of NDCMs involves a high-temperature heat treatment step leading to the formation of random N-containing species. Hence, zeolite-templated porous carbons (ZTCs)^36,37^ have been prepared using zeolites as templates to control the locations of active sites. Carbonization in zeolite pores and removing the zeolite template by a base–acid treatment produce a porous material with a uniform pore size and high surface area. Therefore, ZTCs have been successfully used in various applications, such as the storage of hydrogen and methane, CO_2_ capture,^38^ liquid phase adsorption, catalysis, electrochemical capacitors, batteries, and fuel cells.^36,37,39^

In this study, we applied the ZTC synthesis method to develop highly active NDCMs with high specific surface areas and many N-edge sites. The synthesized NDCMs were used as ORR^13^ catalysts to demonstrate their advantages.

## 2 Experimental

### 2.1 Materials

Protonated zeolites (MFI, FAU, FER, LTL, and MOR) with SiO_2_/Al_2_O_3_ ratios of 23.1, 7.2, 17.6, 6.2, and 18.0, respectively, were purchased from Tosoh Corporation. Glycine and 4 M NaOH, 5 M HCl, and 0.1 M KOH solutions were acquired from Wako Pure Chemical Industries.

### 2.2 Synthesis of NDCMs

First, 0.2 g of glycine was dissolved in water and mixed with 0.2 g of MFI zeolite. The resulting mixture was dried at 90 °C, and the obtained powder (zeolite/glycine precursor) was heated to 1100 °C in a tube furnace under a N_2_ atmosphere at a heating rate of 6 °C min^−1^. The obtained sample (denoted as NC/MFI) was immersed into the 4 M NaOH solution at 90 °C for 16 h to remove silicates from the zeolite structure. The mass ratio between the main components was 1 (NC/MFI)/50 (NaOH). After water filtration, the remaining alumina was removed from the zeolite sample by the treatment with the 5 M HCl solution. The resultant powder was washed with deionized water until the solution was neutral. The obtained sample was denoted as NC. For comparison, only glycine was carbonized under the same conditions to validate the ZTC synthesis method (the obtained sample was labeled Gly).

To examine the effect of glycine addition, the same experiment was repeated at various glycine contents. The samples synthesized at glycine amounts of 0.1, 0.2, and 0.4 g and heated at 1100 °C were labeled MFI-1100-0.1, MFI-1100-0.2, and MFI-1100-0.4, respectively.

In addition, we changed the carbonization temperature without varying the other parameters. The samples synthesized at 900, 1000, and 1100 °C were named MFI-900-0.2, MFI-1000-0.2, and MFI-1100-0.2, respectively.

Finally, we synthesized NCDMs using the other types of zeolites (FAU, FER, LTL, and MOR) *via* a similar procedure. The obtained MFI, FAU, FER, LTL, and MOR samples were denoted as MFI-1100-0.2, FAU-1100-0.2, FER-1100-0.2, LTL-1100-0.2, and MOR-1100-0.2, respectively.

### 2.3 Characterization

X-ray diffraction (XRD) was performed on a PANalytical X'Pert-MDR diffractometer using Cu Kα radiation to determine the crystal structure. The grain sizes and shapes of the prepared samples were observed on a Hitachi H-800 transmission electron microscope. Energy-dispersive X-ray spectroscopy (EDX) was conducted using a JEOL JCM-7000 microscope to confirm the presence of the Si and Al zeolite components. Nitrogen adsorption measurements were performed at 77 K using a Microtrac BELSORP MINI X after heating and vacuum degassing at 523 K for 3 h. The specific surface area and pore size distribution analysis were performed by Brunauer–Emmett–Teller (BET) and Brett–Joyner–Halenda (BJH) methods, respectively. X-ray photoelectron spectroscopy (XPS) was conducted on a JEOL JPS-9000MX spectrometer with Mg Kα radiation (10 kV, 10 mA) serving as the energy source to obtain C 1s and N 1s spectra.

### 2.4 Electrocatalytic activity evaluation for the ORR

The electrochemical activity of the prepared samples was measured in a three-electrode cell using a Model 2325 Bi-Potentiostat (BAS, Japan) attached to a rotating ring disk electrode (RRDE) apparatus (BAS, Japan). An RRDE containing a glassy carbon ring disk (diameter: 4 mm) loaded with each sample was used as the working electrode; a Hg/Hg_2_Cl_2_ (saturated KCl) electrode served as the reference electrode; and a Pt wire was utilized as the counter electrode. A catalyst ink was prepared by dispersing each sample (8.8 mg) in a mixture containing 80 vol% deionized water, 0.02 vol% Nafion® (Aldrich), and 20 vol% isopropanol (Wako). The produced RRDEs were coated with 8 μL of the ink and dried in air. A potential *versus* a saturated calomel electrode (SCE) was converted to a potential *versus* a reversible hydrogen electrode (RHE) *via* the Nernst equation:*E*_RHE_ = *E*_SCE_ + 1.0083

The ORR activity was evaluated by linear sweep voltammetry (LSV) using an RRDE-3A (BAS) electrode in an O_2_-saturated 0.1 M KOH solution in the potential range of 0.2–1.0 V (*vs.* RHE) at a scan rate of 10 mV s^−1^. As a comparison, we used 20 wt% Pt/C (Aldrich).

## 3 Results and discussion

First, the crystal structures of the N-doped zeolite carbon composites were investigated by XRD. The characteristic peaks of MFI zeolite templated carbon (NC/MFI) located at 8.0°, 8.8°, and 23.1° (Fig. S1(a)†) revealed that glycine carbonization occurred while the MFI-type zeolite retained its crystalline structure. In contrast, the composites of N-doped carbon and zeolites obtained from the other zeolites were amorphous (Fig. S1†) because their zeolite frameworks collapsed during the carbonization procedure. Therefore, we can say that MFI zeolite produced the most stable template.

EDX measurements were performed to determine zeolite compositions before and after the base and acid treatments. The presence of Si and Al elements, the main zeolite components, in NC/MFI indicates that NC/MFI is composed of the zeolite template and N-doped carbons (Table 1). After the base-acid treatments, the amounts of Si and Al decrease dramatically. The composites of N-doped carbons and amorphous aluminosilicates derived from the other zeolites are listed in Table S1.† The observed effects of the base and acid treatments varied slightly depending on the zeolite type. In particular, the highest amounts of Si and Al atoms remained even after the FAU zeolite removal treatment because of its high Si/Al ratio and Al content. Meanwhile, MFI zeolite was easiest to remove by the base and acid treatments.

**Table 1:** Relative fractions of Si and Al elements in the NC/MFI and NC samples

Sample	Si [mol%]	Al [mol%]
NC/MFI	26.80	2.69
NC	0.62	n.d.

Tables S2 and S3† list the carbon-to-zeolite mass ratios of the zeolite-N-doped carbon complexes determined by thermogravimetric (TG) analysis, and Tables S4 and S5† present the ratios between the corresponding zeolites and total N-doped carbons.

They show that both ratios increase with increasing glycine amount (Table S2†). In addition, MFI-1100-0.2 exhibits the lowest zeolite content after varying the glycine amount (Table S4†). Note that the carbon/zeolite ratio is independent of the carbonization temperature, indicating that this temperature does not affect the carbon amount (Table S3†). However, it strongly influences the ease of zeolite removal (Table S5†). The higher the carbonization temperature, the larger the amount of removed zeolite because the zeolite structure collapses at higher temperatures and then is easily removed by the base and acid treatments.

**Fig. 2:**
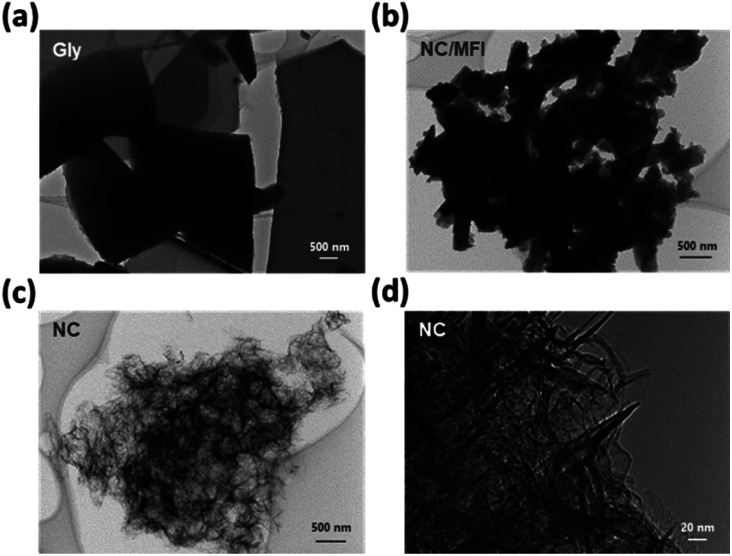
TEM images of (a) Gly, (b) NC/MFI, and (c and d) NC.

The morphology of each sample was examined by transmission electron microscopy (TEM). Gly, which was synthesized by carbonizing glycine alone, was polymerized to form a sheet-like structure (Fig. 2a). NC/MFI possessed a morphology similar to that of the parent MFI zeolite, indicating that glycine was polymerized and carbonized within zeolitic micropores (Fig. 2b). Meanwhile, pores were formed in the NC sample after the zeolite removal treatment (Fig. 2c), while NC exhibited a fibrous structure (Fig. 2d). These results indicate that the zeolite template method can prevent glycine from polymerizing into a sheet-like structure. Furthermore, the zeolite template was effectively removed by the base and acid treatments, which enabled the synthesis of the porous N-doped carbon material. According to Fig. S3,† the carbon content increases with an increase in glycine amounts. Moreover, sheet-like structure carbon was observed on the surface of the MFI-1100-0.4 sample, suggesting that its glycine content was excessive. Notably, the zeolite pores were filled in the composite structure regardless of the zeolite type, while after the base and acid treatments, the zeolite template was removed, leading to the formation of the pore (Fig. S4†).

**Fig. 3:**
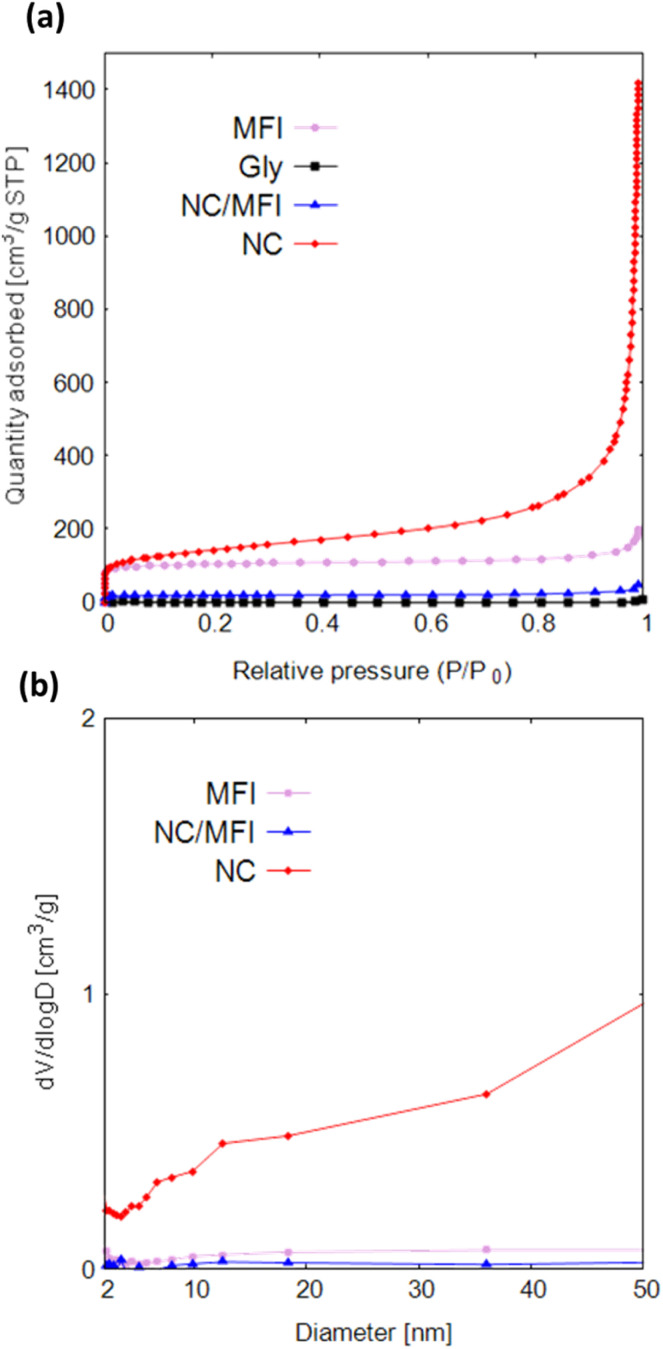
(a) N_2_ adsorption isotherms and (b) pore size distributions of MFI, Gly, NC/MFI, and NC.

Fig. 3a and Table S6† show that Gly does not adsorb nitrogen or contains pores. The adsorption capacity of NC/MFI is much lower than that of MFI, suggesting that glycine penetrated the pores of the MFI-type zeolite and was carbonized inside the pores. Both the adsorption amount and specific surface area of NC are considerably larger than those of MFI, NC/MFI, and Gly. This result indicates that the synthesis performed using zeolite as a template is accompanied by pore formation, which increases the specific surface area.

The NC isotherm spanning over the entire pressure region indicates that pores of various sizes (ranging from micropores to macropores) were formed in the NC sample. Due to this, NC showed much higher values in wide mesopore size regions than NC/NFI and MFI (Fig. 3b). Fig. S5a and Table S6† show that with an increase in the glycine amount, the specific surface area and the number of pores decrease. Fig. S6a† also reveals that MFI-1100-0.1 contains more mesopores. Furthermore, MFI-1100-0.4 has fewer mesopores and macropores than MFI-1100-0.2. According to Fig. S5b and Table S6,† the specific surface areas of various zeolites decrease in the order of FAU, MFI, LTL, MOR, and FER. As shown in Fig. S6b,† FAU-1100-0.2 contains fewer mesopores than MFI-1100-0.2.

**Fig. 4:**
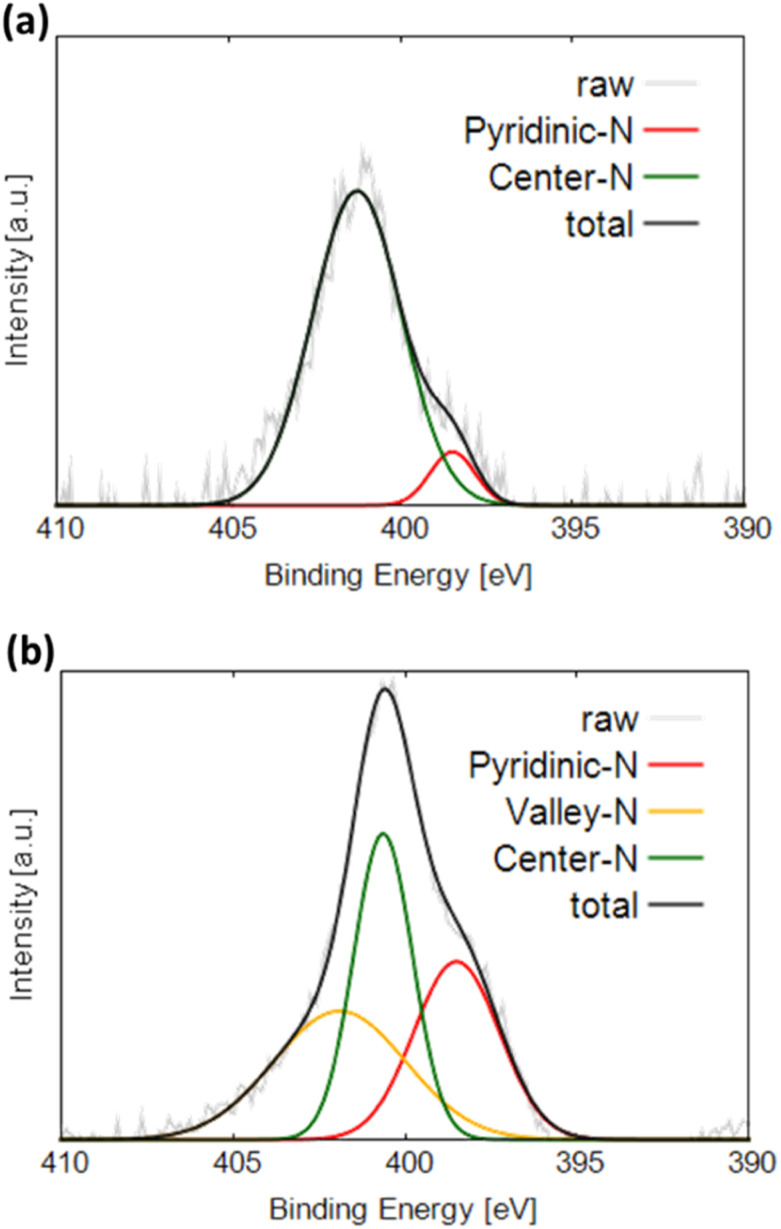
N 1s XPS profiles of (a) Gly and (b) NC.

Fig. 4a and b display the N 1s XPS profiles of NC and Gly, respectively. The obtained spectra are fitted with three Gaussian functions corresponding to different nitrogen components and labeled pyridinic-N (398.5 eV), center-N (401.0 eV), and valley-N (401.9 eV).^21,30^ Pyridinic-N is bonded to two carbon atoms and located at graphite edges or inside the pores. Center-N and valley-N sites are bound to three carbon atoms. Center-N is present on the graphite basal plane, and valley-N is located at graphite edges or inside the pores. Both NC and Gly exhibit the highest fractions of center-N sites. On the one hand, NC produces the pyridinic-N and valley-N peaks in addition to the center-N peak. On the other hand, only the pyridinic-N peak and center-N peak were observed in Gly. The valley-N peak cannot be observed for Gly because its intensity is too low to be separated from the central N peak, suggesting that Gly may also contain a small amount of valley-N sites.

Note that the pyridinic-type nitrogen, which is more electronegative than carbon, is negatively charged. This likely results in the flow of N π-electrons into the p_z_-orbitals of the adjacent carbon atoms, which become basic sites and thus can effectively adsorb oxygen species. In contrast, the π electrons of the central nitrogen sites flow into the graphene structure containing stable π-conjugated systems around nitrogen atoms and become delocalized.^21^ Therefore, the p_z_-orbitals of the carbon atoms adjacent to the nitrogen atoms remain unoccupied and do not form basic sites, preventing the adsorption of oxygen molecules.^22^ The valley nitrogen atoms, which are not surrounded by a stable π-conjugated system, are similar to the pyridinic nitrogen atoms and expected to form basic sites, which effectively adsorb oxygen at the initial ORR stages.

**Fig. 5:**
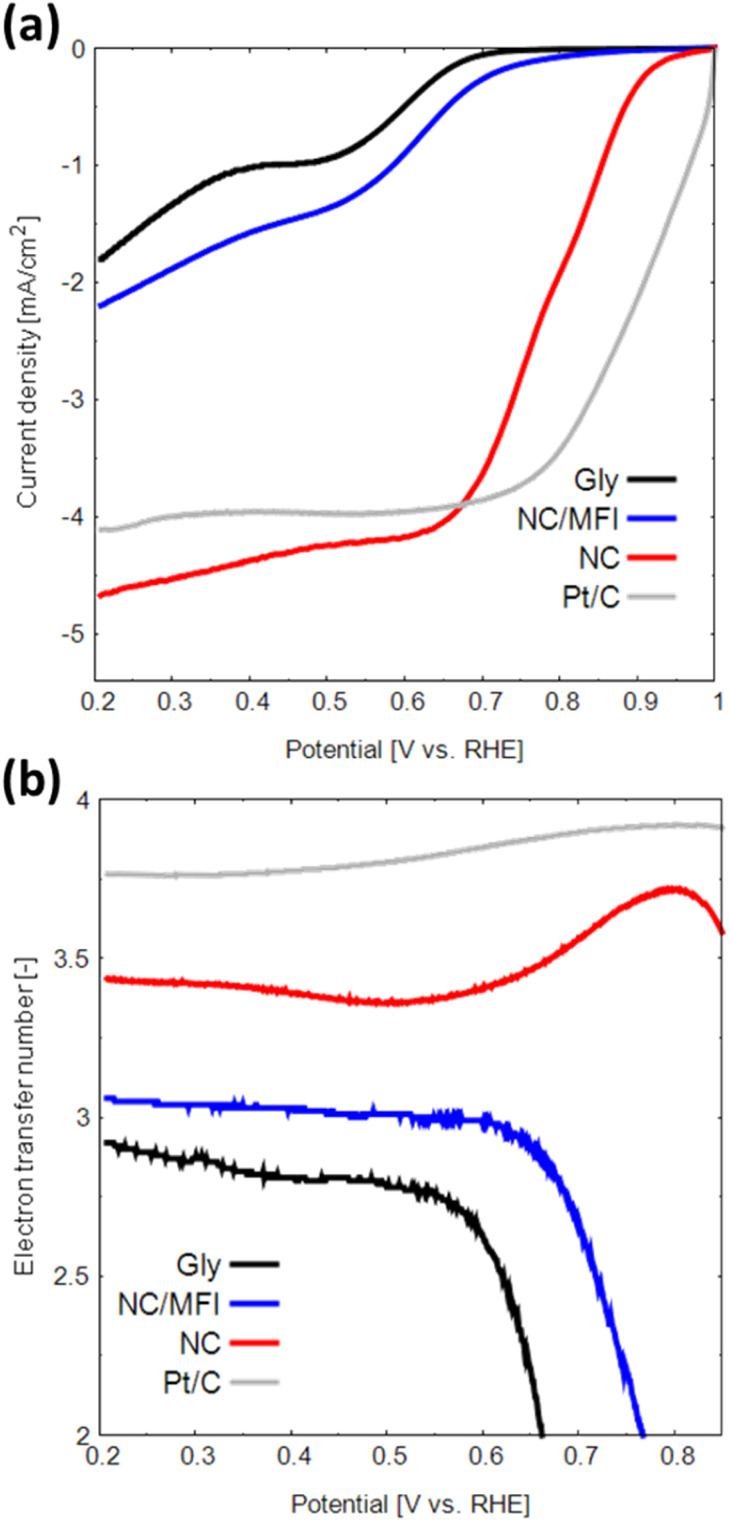
(a) LSV curves and (b) numbers of transferred electrons obtained for Gly, NC/MFI, NC and Pt in the oxygen-saturated 0.1 M KOH solution at a scan rate of 5 mV s^−1^.

LSV measurements were performed to evaluate the ORR performance of the N-doped carbon materials. As shown in Fig. 5a, the onset potential, half-wave potential, and limiting current density of NC are considerably higher than those of Gly (see Table 2). In addition, NC showed a comparable current density at 0.7 V *vs.* RHE to a Pt-based catalyst. This result indicates that the N-doped carbon materials synthesized using zeolite as a template are catalytically active. NC also demonstrates higher performance than that of NC/MFI, confirming the effectiveness of zeolite removal by the base and acid treatments.

**Table 2:** Onset potential (*E*_onset_), half-wave potential (*E*_half-wave_), and limiting current densities of Gly, NC/MFI, and NC

Sample	*E*_onset_ [V *vs.* RHE]	*E*_half-wave_ [V *vs.* RHE]	Limiting current density [mA cm^−2^]
Gly	0.68	0.52	−1.80
NC/MFI	0.72	0.57	−2.20
NC	0.90	0.78	−4.67

The H_2_O_2_ yield and number of transferred electrons during the ORR were determined from the obtained LSV data. The two main types of ORRs include two-electron and four-electron reactions.^23,40,41^ The two-electron reaction generates hydrogen peroxide, which causes catalyst degradation and produces a low electromotive force. In contrast, the four-electron reaction proceeds in a single step and generates a high electromotive force.

The H_2_O_2_ generation rate of NC is 14–32%, which is lower than that of Gly (Fig. S8a†). The number of transferred electrons in NC is approximately 3.38–3.75 (Fig. 5b), indicating that the four-electron reaction proceeds at a relatively high rate. Thus, the N-doped carbon material synthesized using a zeolite template exhibits high performance as an ORR catalyst for the following three reasons. First, the high catalytic activity of N-doped carbon can be attributed to a large number of active sites due to the large specific surface area. Second, the macropores formed in the NC structure promote the diffusion of oxygen, the main ORR reactant. Finally, the formation of N-doped carbon with multiple edge sites increases the numbers of catalytically active pyridinic-N and valley-N sites.^40^ The observed effect can potentially improve the catalytic performance of N-doped carbon materials in other reactions besides the ORR.

Finally, the ORR catalytic properties of the samples synthesized at different glycine amounts, carbonization temperatures, and zeolite types were examined. The optimal glycine amount is 0.2 g (Fig. S7a†) because the catalyst pores become filled and the number of edge sites decreases at excessive glycine contents. Conversely, when the glycine amount is insufficient, macropores are formed less frequently. The carbonization temperature does not significantly affect the ORR performance, although the onset potential of MFI-1100-0.2 is slightly higher than those of MFI-1000-0.2 and MFI-900-0.2 (Fig. S7b†). The onset potentials measured for different types of zeolites decrease in the order of MFI-1100-0.2, FAU-1100-0.2, FER-1100-0.2, LTL-1100-0.2, and MOR-1100-0.2 (Fig. S7c†). The ORR activity for different glycine amounts, carbonization temperatures, and zeolite types could also be discussed from the H_2_O_2_ yield and number of transferred electrons (Fig. S8 and S9†). These results indicate that N-doped carbon materials with high ORR activities can be obtained when zeolites with high-dimensional pore alignment are used as templates because they easily form defects after zeolite removal and the introduction of active sites. In addition, the ORR catalytic properties of all samples synthesized using different zeolite templates were superior to those of Gly, indicating that the proposed zeolite templating method enhanced the ORR performance of N-doped carbons.

Finally, the chemical resistance was examined, and it was found that N-doped carbon materials synthesized using a zeolite template are more resistant to chemicals than a platinum catalyst (Fig. S10†). This higher chemical resistance is an additional attraction of the metal-free N-doped carbon material.

## 4 Conclusion

In this study, N-doped carbon catalysts were synthesized by carbonizing amino acids using zeolites as templates. The catalyst obtained using MFI zeolite exhibited a high specific surface area and ORR activity corresponding to an onset potential of 0.90 V *vs.* RHE and a current density of −4.67 mA cm^−2^. The prepared N-doped carbon sample also contained large numbers of pyridinic-N and valley-N sites, which served as the active sites for the ORR. Moreover, the zeolite templating method allowed selective doping of carbon matrices with nitrogen atoms at the edge sites of graphite. This work describes a new application of the zeolite templating method in addition to pore formation.

## Author contributions

Y. T. and K. M performed the experiments, comprehensive study and manuscript drafting. These two authors contributed equally to this work. Y. S. and R. T. performed the experiments and developed the methodology. Y. U., Y. S. and R. T. performed the writing (review & editing). K. M. and N. N. supervised this work.

## Conflicts of interest

There are no conflicts to declare.

## Supplementary Material

NA-005-D3NA00186E-s001
