# Supplementary material for: A zeolite templating method for fabricating edge site-enriched N-doped carbon materials
Source: Nanoscale Adv. 2023 May 12;5(16):4233–9. doi: 10.1039/d3na00186e (PMC10408580; doi:10.1039/d3na00186e)
Supplement: NA-005-D3NA00186E-s001 [file NA-005-D3NA00186E-s001.pdf]

Electronic Supplementary Material (ESI) for Nanoscale Advances.
This journal is © The Royal Society of Chemistry 2023

Supporting Information

A zeolite templating method for fabricating edge site-enriched N-doped carbon materials

Yurika Taniguchi [a], *Yasuhiro Shu* [a], *Ryuji Takada* [a], *Koji Miyake* * [a,b], *Yoshiaki Uchida* [a], *Norikazu Nishiyama* [a,b]

[a] Division of Chemical Engineering, Graduate School of Engineering Science Osaka University 1-3 Machikaneyama, Toyonaka, Osaka 560-8531, Japan
[b] Innovative Catalysis Science Division, Institute for Open and Transdisciplinary Research Initiatives (ICS-OTRI), Osaka University, Suita, Osaka 565-0871, Japan.

*Corresponding author: kojimiyake@cheng.es.osaka-u.ac.jp

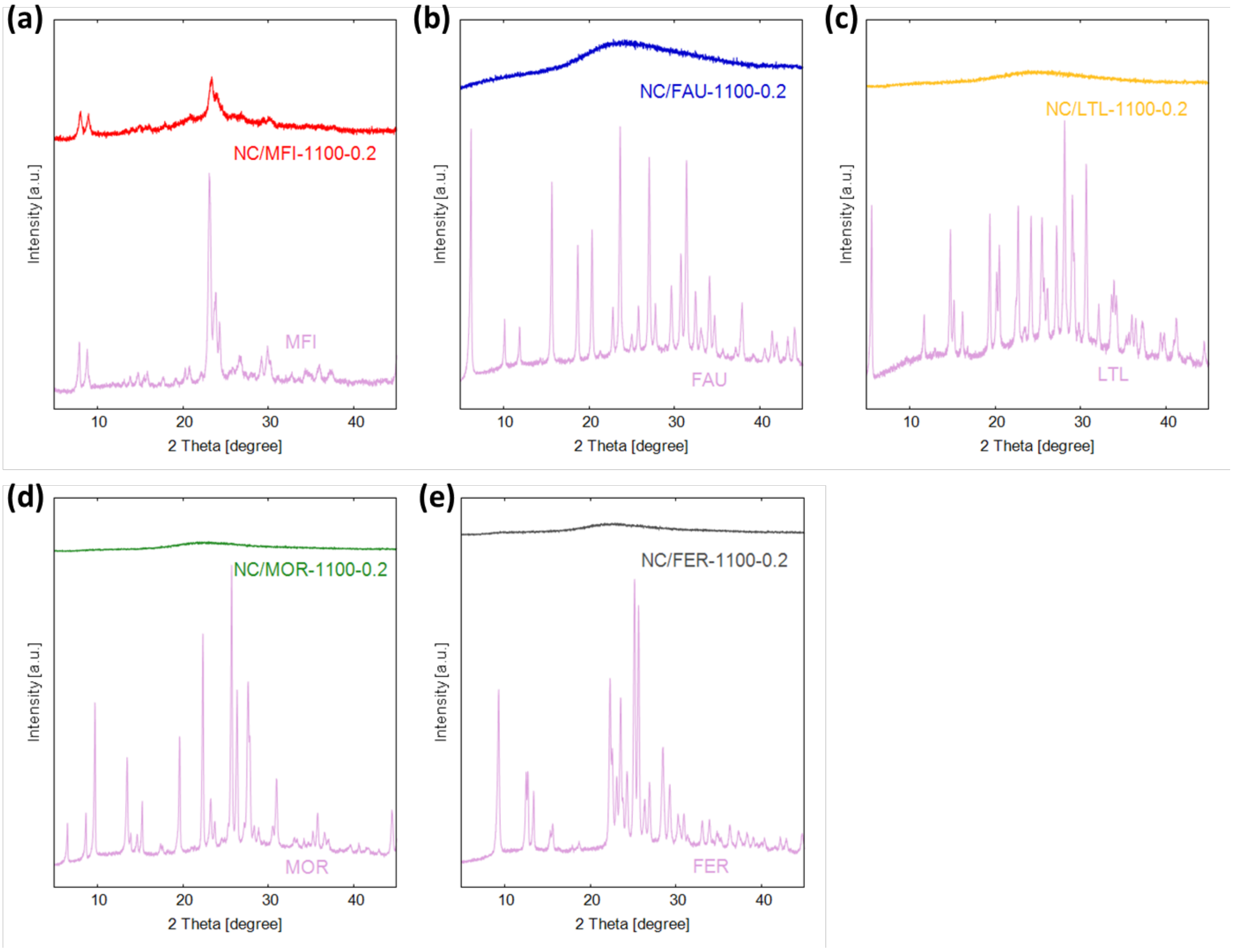

Figure S1. XRD pattern of (a) NC/MFI-1100-0.2 (b) NC/FAU-1100-0.2 (c) NC/LTL-1100-0.2 (d) NC/MOR-1100-0.2 and (e) NC/FER-1100-0.2.

Table S1. Percentage of Si and Al in samples synthesized using various types of zeolites as templates analyzed by EDX.

Sample	Si [mol %]	Al [mol %]
NC/MFI-1100-0.2	26.80	2.69
MFI-1100-0.2 (= NC)	0.62	n.d.
NC/FAU-1100-0.2	18.14	6.93
FAU-1100-0.2	1.15	0.35
NC/LTL-1100-0.2	15.66	5.59
LTL-1100-0.2	0.45	0.06
NC/MOR-1100-0.2	23.13	2.74
MOR-1100-0.2	0.30	0.11
NC/FER-1100-0.2	24.94	2.70
FER-1100-0.2	0.32	0.16

Table S2. The ratio of carbon to zeolite in the zeolite N-doped carbon (NC/MFI-1100-0.1, NC/MFI-1100-0.2 and NC/MFI-1100-0.4) complex analyzed by TG.

Sample	Mass ratio of carabon/zeolite [-]
NC/MFI-1100-0.1	0.063
NC/MFI-1100-0.2 (= NC)	0.12
NC/MFI-1100-0.4	0.38

Table S3. The ratio of carbon to zeolite in the zeolite N-doped carbon (NC/MFI-900-0.2, NC/MFI-1000-0.2 and NC/MFI-1100-0.2) complex analyzed by TG.

Sample	Mass ratio of carabon/zeolite [-]
NC/MFI-900-0.2	0.17
NC/MFI-1000-0.2	0.15
NC/MFI-1100-0.2 (= NC)	0.12

Table S4. The ratio of zeolite to total N-doped carbon (MFI-1100-0.2, MFI-1000-0.2 and MFI-1100-0.2) complex analyzed by TG.

Sample	Mass ratio of zeolite/total [-]
NC/MFI-1100-0.1	0.061
NC/MFI-1100-0.2 (= NC)	n.d.
NC/MFI-1100-0.4	0.055

Table S5. The ratio of zeolite to total N-doped carbon (MFI-900-0.2, MFI-1000-0.2 and MFI-1100-0.2) complex analyzed by TG.

Sample	Mass ratio of zeolite/total [-]
NC/MFI-900-0.2	0.13
NC/MFI-1000-0.2	0.041
NC/MFI-1100-0.2 (= NC)	n.d.

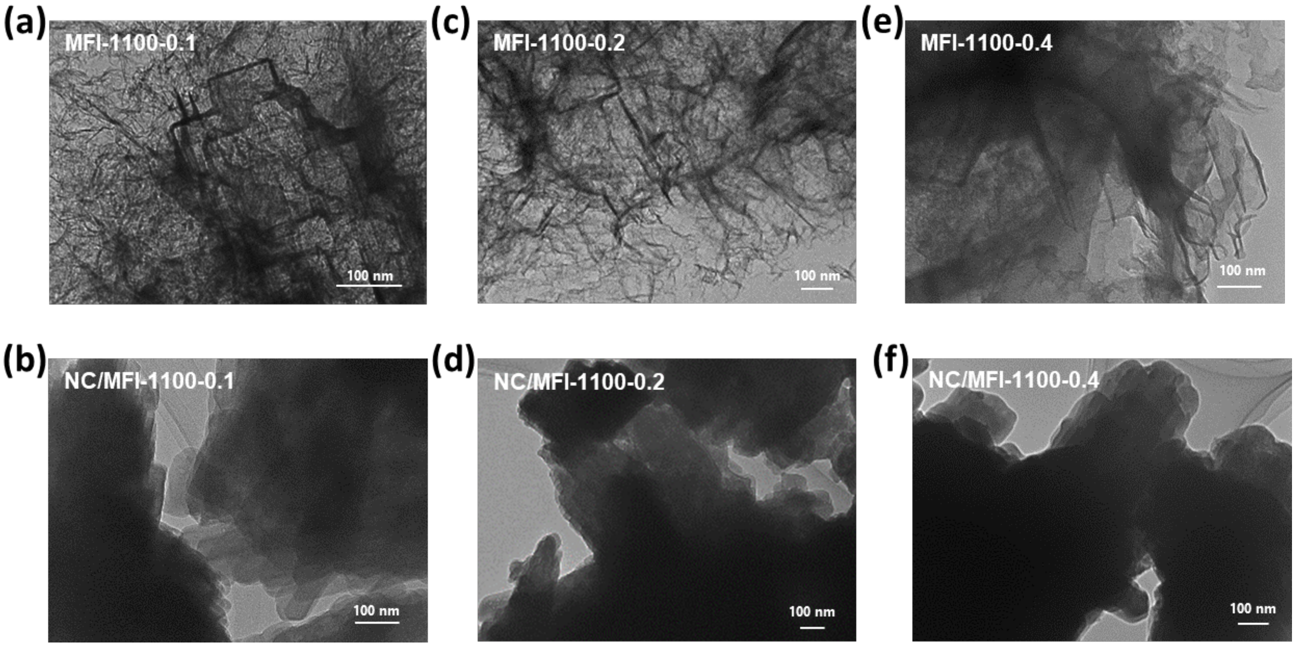

Figure S3. TEM images of samples before and after basic acid treatment when synthesized with different amounts of glycine: (a) (b) 0.1g, (c) (d) 0.2g, and (e) (f) 0.4g.

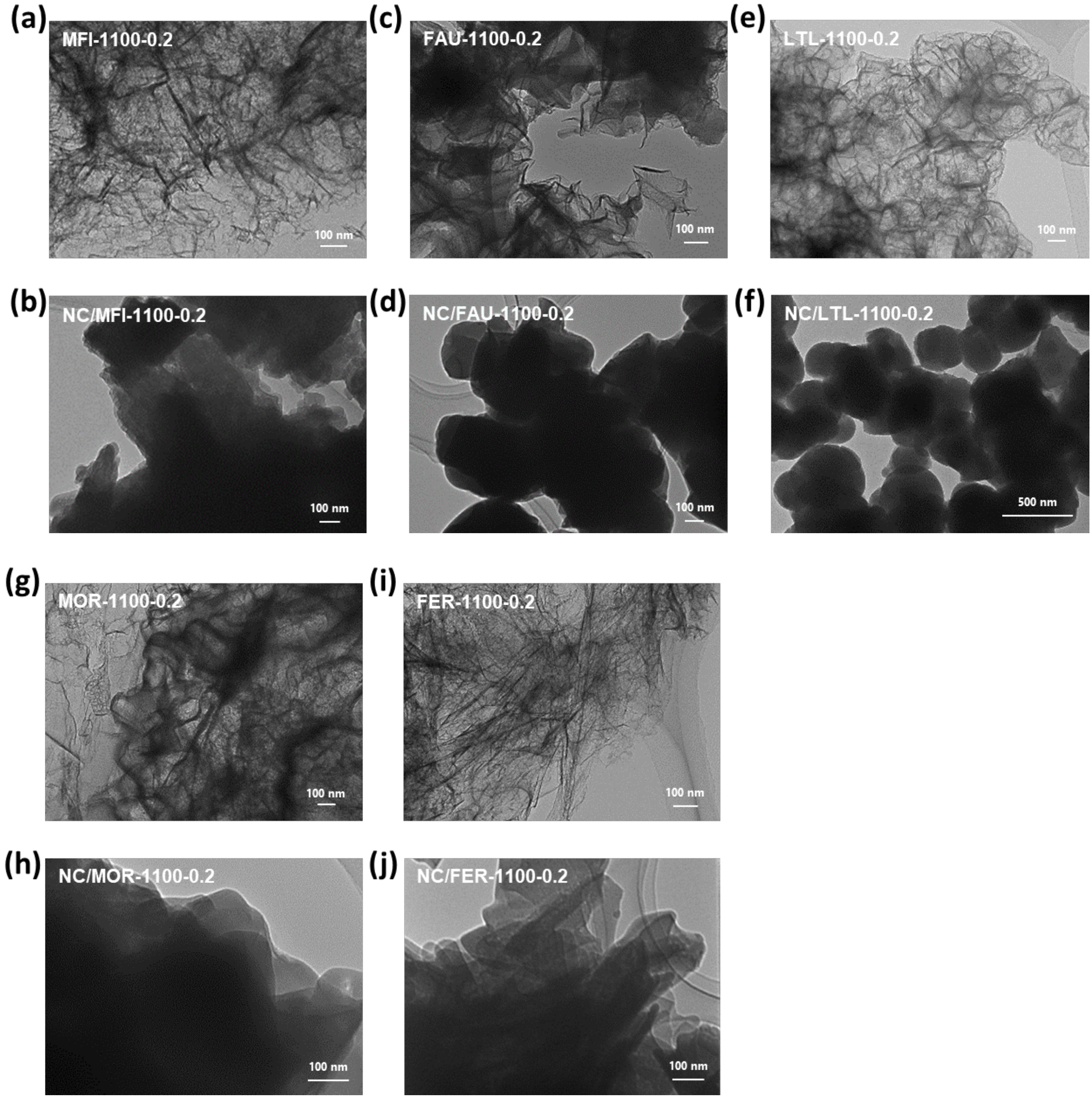

Figure S4. TEM images of samples before and after basic acid treatment with different types of zeolite as a template: (a) (b)MFI, (c) (d) FAU, (e) (f) LTL, (g) (h) MOR, and (i) (j) FER.

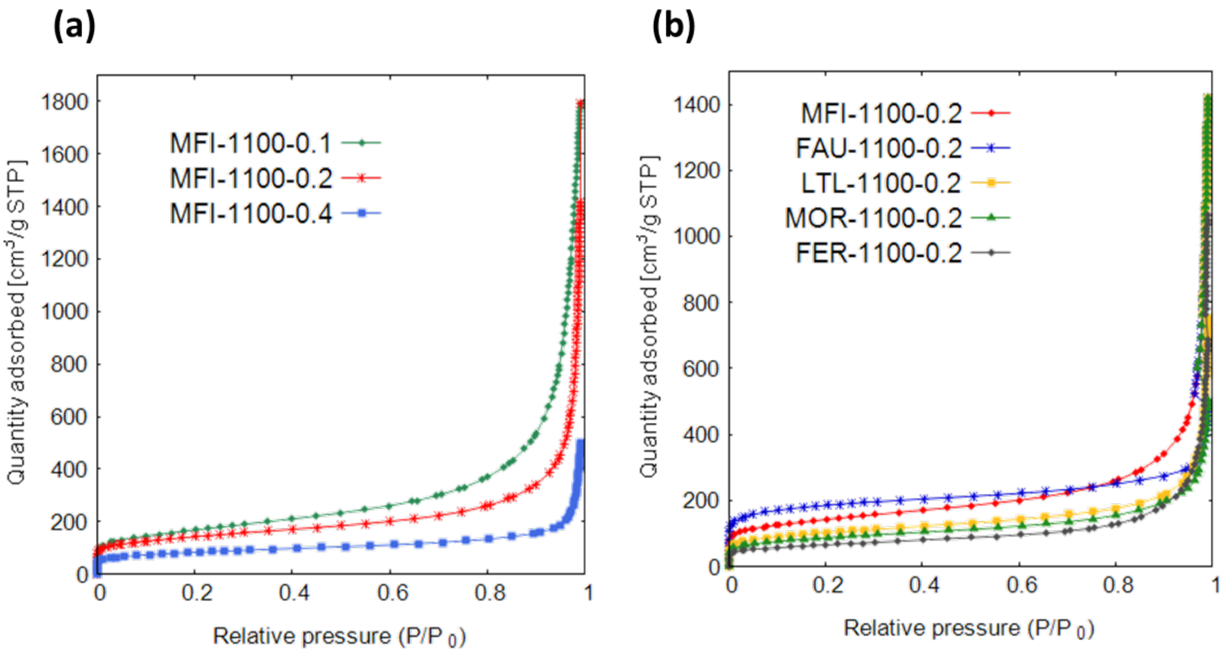

Figure S5. N_2 adsorption isotherms of (a) samples synthesized with different amounts of glycine (0.1, 0.2, and 0.4 g) and (b) samples synthesized with different types of zeolite (MFI, FAU, LTL, MOR, and FER).

Table S6. The specific surface area of main samples (MFI, Gly, NC/MFI, and NC), samples synthesized with different amounts of glycine (0.1, 0.2, and 0.4 g) and samples synthesized with different types of zeolite (MFI, FAU, LTL, MOR, and FER).

Sample	S_{BET} [m^2/g]
MFI	399
Gly	0
NC/MFI	73
NC	507
MFI-1100-0.1	595
MFI-1100-0.2 (= NC)	507
MFI-1100-0.4	297
MFI-1100-0.2 (= NC)	507
FAU-1100-0.2	678
LTL-1100-0.2	369
MOR-1100-0.2	312
FER-1100-0.2	233

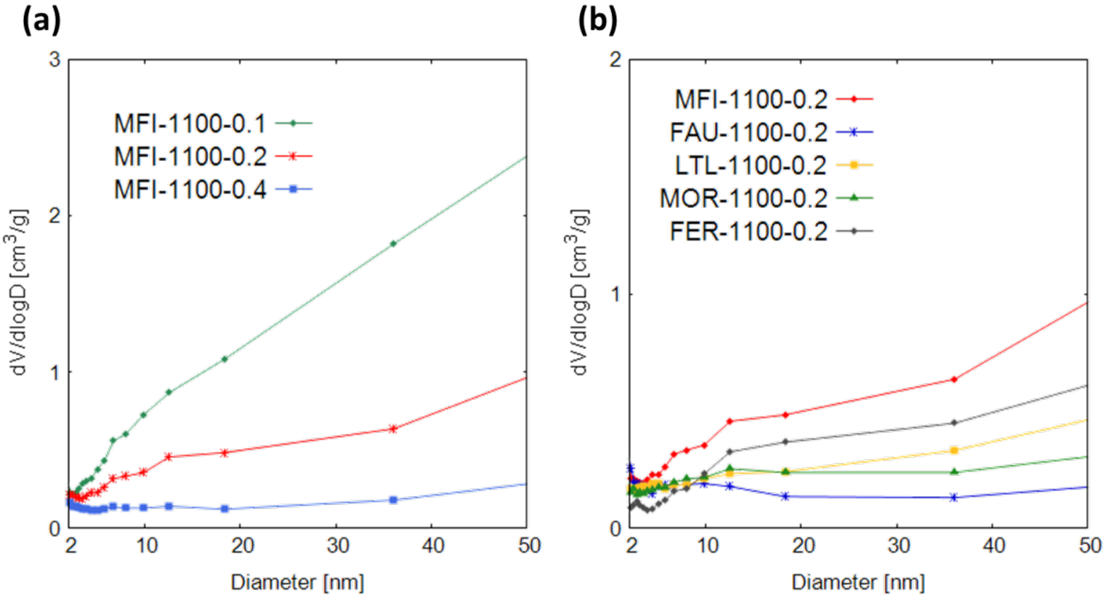

Figure S6. Pore size distribution of (a) samples synthesized with different amounts of glycine (0.1, 0.2, and 0.4 g) and (b) samples synthesized with different types of zeolite (MFI, FAU, LTL, MOR, and FER).

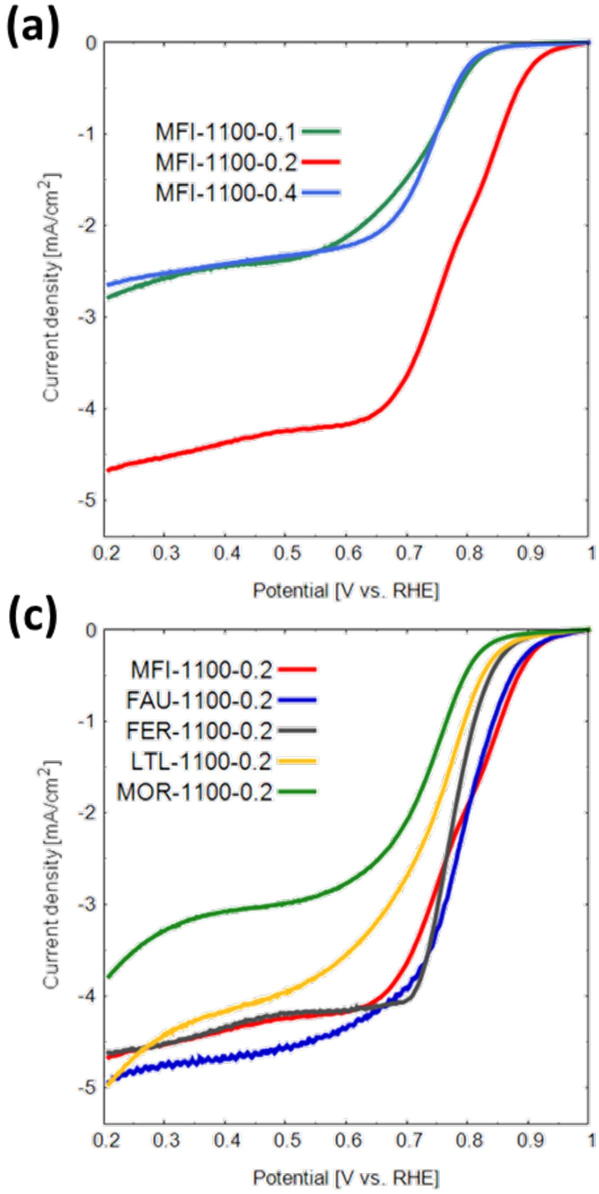

Figure S7. LSV curves of (a) samples synthesized with different amounts of glycine (0.1 , 0.2 , and 0.4 g), (b) samples synthesized with different carbonization temperatures (900, 1000, and 1100 °C) and (c) samples synthesized with different types of zeolite (MFI, FAU, LTL, MOR, and FER).

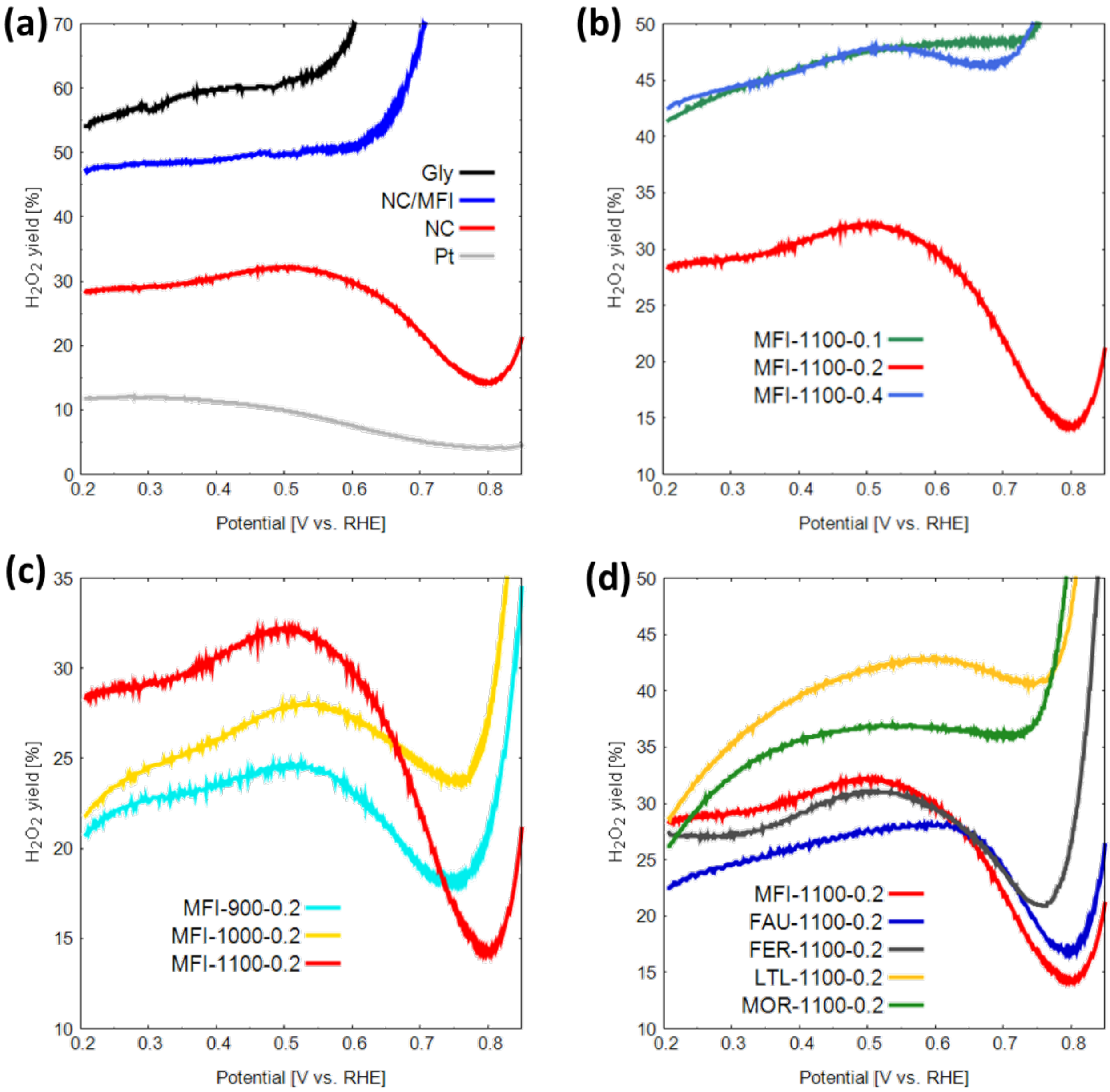

Figure S8. H_2O_2 yield of (a) main samples (Gly, NC/MFI, NC and Pt), (b) samples synthesized with different amounts of glycine (0.1, 0.2, and 0.4 g), (c) samples synthesized with different carbonization temperatures (900, 1000, 1100 ^{o}C) and (d) samples synthesized with different types of zeolites (MFI, FAU, LTL, MOR, and FER).

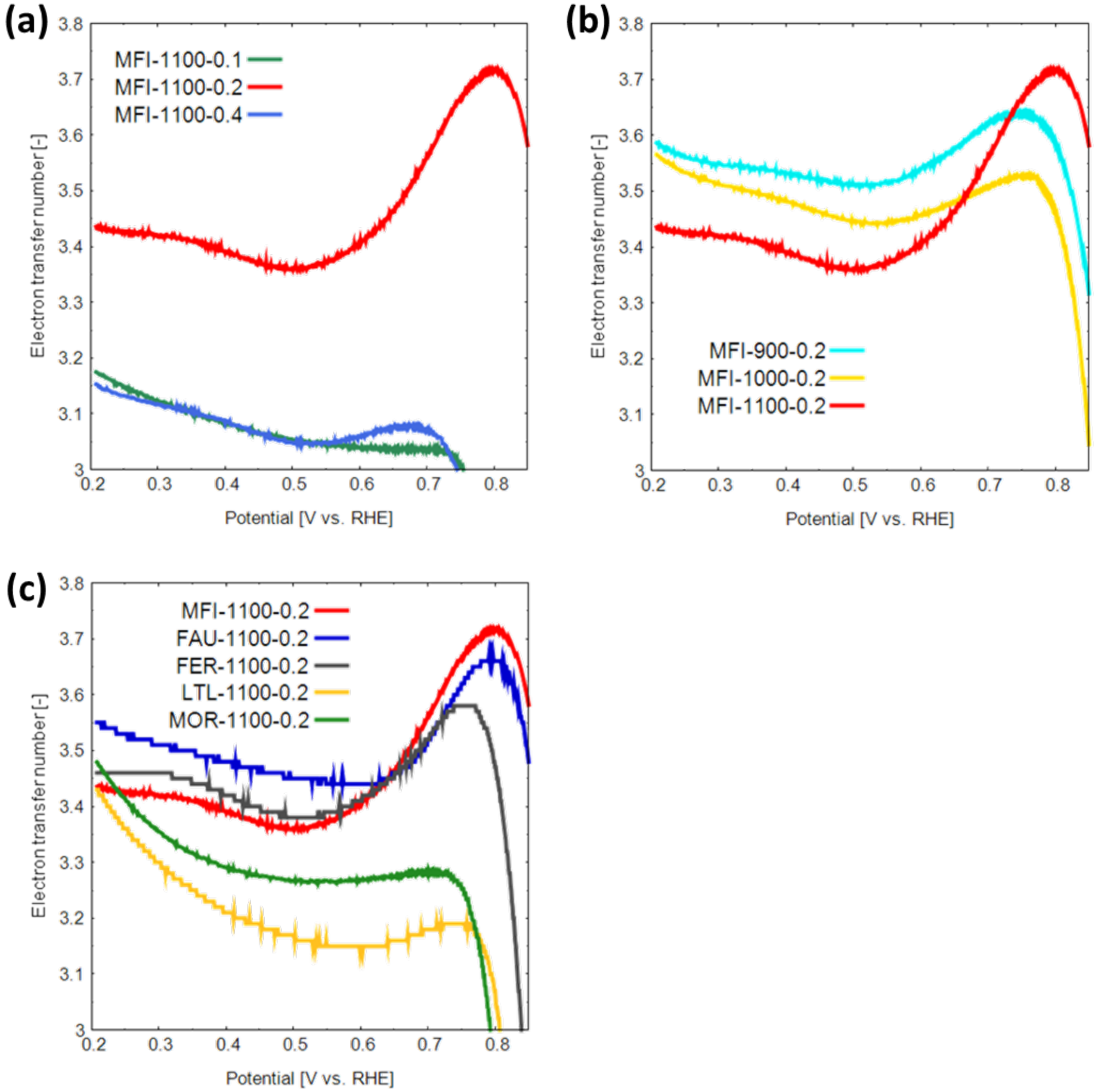

Figure S9. The number of transferred electrons of (a) samples synthesized with different amounts of glycine (0.1, 0.2, and 0.4 g), (b) samples synthesized with different carbonization temperature (900, 1000, and 1100 ^{o}C) and (c) samples synthesized with different types of zeolites (MFI, FAU, LTL, MOR, and FER).

Table S7. Onset potential (E_{onset}), half-wave potential ($E_{half-wave}$) and limiting current density of samples synthesized with different amounts of glycine (0.1, 0.2, and 0.4 g), samples synthesized with different carbonization temperature (900, 1000, and 1100 ºC) and samples synthesized with different types of zeolite (MFI, FAU, LTL, MOR, and FER).

Sample	E_{onset} [V vs. RHE]	$E_{half-wave}$ [V vs. RHE]	Limiting current density [mA/cm^2]
MFI-1100-0.1	0.82	0.71	-2.78
MFI-1100-0.2 (= NC)	0.90	0.78	-4.67
MFI-1100-0.4	0.81	0.73	-2.65
MFI-900-0.2	0.89	0.76	-4.23
MFI-1000-0.2	0.86	0.76	-4.86
MFI-1100-0.2 (= NC)	0.90	0.78	-4.67
MFI-1100-0.2 (= NC)	0.90	0.78	-4.67
FAU-1100-0.2	0.88	0.79	-4.95
FER-1100-0.2	0.84	0.77	-4.61
LTL-1100-0.2	0.84	0.72	-4.96
MOR-1100-0.2	0.82	0.72	-3.79

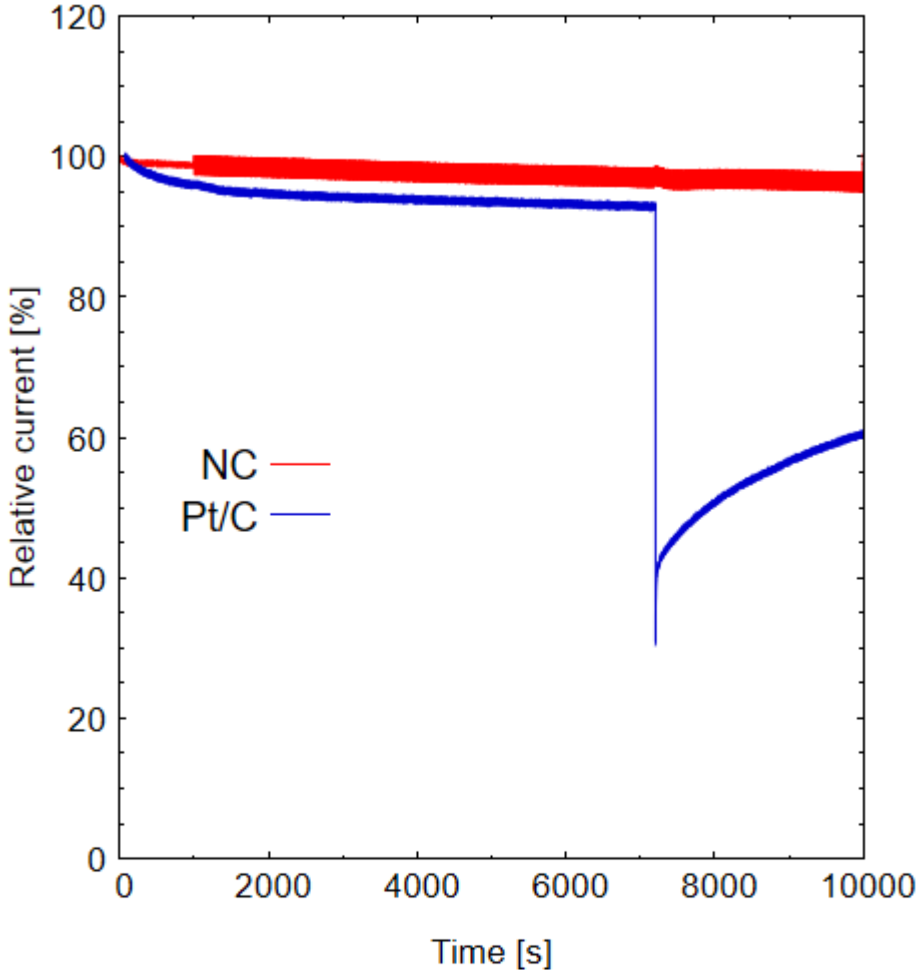

Figure S10. Relative current versus time for NC and Pt/C at 0.6 V vs. RHE with addition of 1 M methanol after 7200 s.

Table S8. The elemental analysis results of samples synthesized with different amounts of glycine (0.1, 0.2, and 0.4 g) and samples synthesized with different types of zeolite (MFI, FAU, LTL, MOR, and FER).

Sample	C [wt %]	H [wt %]	N [wt %]
MFI-1100-0.1	74.98	1.78	3.76
MFI-1100-0.2 (= NC)	76.92	0.98	3.41
MFI-1100-0.4	74.82	1.96	3.40
MFI-1100-0.2 (= NC)	76.92	0.98	3.41
FAU-1100-0.2	62.26	2.83	4.93
FER-1100-0.2	79.71	1.65	4.33
LTL-1100-0.2	71.19	1.92	3.81
MOR-1100-0.2	77.76	1.71	4.41

Table S9. Comparison of the electrocatalytic activities of catalysts in O_2-saturated 0.1 M KOH solution. (All data were obtained via LSV at a 1600 rpm rotational speed).

Catalyst	E_{onset} [V vs. RHE]	Limiting current density [mA/cm2]	Reference
NC	0.90	-4.67	This work
PANI_0.2_1000_N2_1200	0.97	-5.80	J. Mater. Chem. A, 2019, 7, 24239-24250
AN	0.82	-4.30	Catalysis Today, 2019, 337, 102-109
PNCNs-900	0.88	-4.40	Journal of Alloys and Compounds, 2022, 914, 165359
N-C-900	0.92	-4.19	International Journal of Low-Carbon Technologies, 2020, 17, 1029-1035
N/POPQ800	0.79	-2.10	ACS Appl. Energy Mater, 2022, 5, 15899-15908
